# Preparation of Antimicrobial Agents: From Interpolyelectrolyte Complexes to Silver-Containing Metal–Polymer Complexes and Nanocomposites

**DOI:** 10.3390/polym16192842

**Published:** 2024-10-08

**Authors:** Dmitry I. Klimov, Alexey A. Zharikov, Elena A. Zezina, Elena A. Kotenkova, Elena V. Zaiko, Dagmara S. Bataeva, Anastasia A. Semenova, Yulia K. Yushina, Aleksander A. Yaroslavov, Alexey A. Zezin

**Affiliations:** 1 Department of Chemistry, M.V. Lomonosov Moscow State University, Leninskie gory 1-3, Moscow 119991, Russia; klimovdi@ispm.ru (D.I.K.); garikov-aleksey@mail.ru (A.A.Z.); zezi-len@yandex.ru (E.A.Z.); yaroslav@belozersky.msu.ru (A.A.Y.); 2 Enikolopov Institute of Synthetic Polymeric Materials, Russian Academy of Sciences, Profsoyuznaya st. 70, Moscow 117393, Russia; 3 V.M. Gorbatov Federal Research Center for Food Systems, Talalikhina st., 26, Moscow 109316, Russia; lazovlena92@yandex.ru (E.A.K.); e.zaiko@fncps.ru (E.V.Z.); d.bataeva@fncps.ru (D.S.B.); a.semenova@fncps.ru (A.A.S.); yu.yushina@fncps.ru (Y.K.Y.)

**Keywords:** polyelectrolytes, macromolecular complexes, silver, nanoparticles, bacterial cells, antimicrobial effect

## Abstract

In order to control pathogenic microorganisms, three polymer compositions were prepared and tested. First, a water-soluble positively charged polycomplex was synthesized via the electrostatic binding of anionic polyacrylic acid to an excess of polyethylenimine to enhance the biocidal activity of the polycation. Second, an aqueous solution of AgNO_3_ was added to the polycomplex, thus forming a ternary polycation-polyanion-Ag^1+^ complex with an additional antimicrobial effect. Third, the resulting ternary complex was subjected to UV irradiation, which ensured the conversion of Ag^1+^ ions into Ag nanoparticles ranging in size mainly from 10 to 20 nm. Aqueous solutions of the polymer compositions were added to suspensions of the Gram-positive bacteria *S. aureus* and the Gram-negative bacteria *P. aeruginosa*, with the following main results: (a) Upon the addition of the binary polycomplex, 30% or more of the cells survived after 20 h. (b) The ternary complex killed *S. aureus* bacteria but was ineffective against *P. aeruginosa* bacteria. (c) When the ternary complex with Ag nanoparticles was added, the percentage of surviving cells of both types did not exceed 0.03%. The obtained results are valuable for the development of antibacterial formulations.

## 1. Introduction

Currently, high priority is given to the control of organized communities of microorganisms, while a promising approach to the production of biocidal materials is the synthesis of polymers with cationic groups [1,2,3,4]. Cationic polymers (polycations) bind to the negatively charged cell surface and initiate various processes in the cell membrane, which results in its destruction and, finally, the death of cells [5]. Importantly, the use of polycations does not lead to adaptive effects in bacteria, unlike antibiotics [1,2,5]. Electrostatic complexation of polycations with anionic polymers (polyanions) leads to the formation of interpolyelectrolyte complexes (IPECs), which are a convenient basis for producing biocidal formulations [6,7,8,9,10]. Considerable efforts have been devoted to the development of prolonged antibacterial preparations. It has been shown that metal–polymer conjugates are capable of the controlled release of encapsulated biologically active substances into the environment [2,11,12,13,14,15]. Silver ions and colloids are promising agents for bacterial destruction [8,9,16,17,18,19]. The functional groups of polymers effectively bind to metal ions and stabilize metal nanostructures [13,14,20,21]. IPECs were used as matrices to synthesize metal–polymer complexes and nanocomposites with controlled chemical and supramolecular structures [22,23,24].

IPECs from polyacrylic acid (PAA) and polyethylenimine (PEI) are unique complexing agents capable of binding up to 50 wt% of metal ions due to the formation of ternary complexes with metal ions and carboxylic and nitrogen-containing groups [23]. Previously, stable metal nanoparticles in thin films and coatings were obtained via radiation-induced reduction in metal ions pre-bound to PAA-PEI complexes [8,17,21,25]. There is another easier way to produce biocidal metal nanoparticles: ultraviolet treatment of metal–polymer solutions. UV irradiation is especially promising since it results in metal–polymer nanocomposites without impurities [26,27].

Extensive efforts by our team have been focused on the study of interpolyelectrolyte complexes and functional materials based on them [8,17,21,22,23,25]. Commercially available samples of PAA and branched PEI were used in this work, which opens up prospects for the development of biocidal formulations. To ensure their own antibacterial properties, the complexes contained an excess of cationic PEI. This work aimed at developing methods for the production of biocidal materials. The study of metal–polymer complexes and nanocomposites has shown relevant preliminary results indicating the decisive influence of silver ions and nanoparticles on the antibacterial properties of solutions containing a cationic polymer. Triple metal–polymer complexes and nanocomposites based on the PAA-PEI scaffolds were obtained, and their comparative antibacterial properties were studied for the first time. Taking this into account, in the current article, PEI-PAA IPECs were prepared and then complexed with Ag^1+^ ions, and, finally, the silver ions in the PEI-PAA—Ag^1+^ ternary complex were reduced to Ag nanoparticles (AgNPs). A comparative study of the physico-chemical and biological properties of the three samples, the IPEC, IPEC-Ag^1+^, and IPEC-AgNPs, was undertaken, which allowed us to propose the optimal combination of colloidal and antimicrobial characteristics of metal–polymer formulations.

## 2. Materials and Methods

### 2.1. Reagents

The following reagents were used to prepare the samples: polyacrylic acid (M_w_ = 100,000) from Sigma-Aldrich (St. Louis, MO, USA), polyethylenimine (M_w_ = 60,000) from Serva (Heidelberg, Germany), and silver nitrate of analytical grade from Reachim (Moscow, Russia). The preparation of dispersions of interpolyelectrolyte complexes was discussed in detail in [8]. In order to obtain metal–polymer complexes, a 0.054 wt% solution of AgNO_3_ was added to a 0.2 wt% dispersion of the PEI-PAA IPEC with a molar ratio of cationic PEI and anionic PAA units Q = [PEI]/[PAA] = 3 in low-light conditions with careful stirring, after which the pH values were adjusted to 6 using 0.1 M KOH or H_2_SO_4_ solutions (both manufactured by Reachim, Moscow, Russia).

### 2.2. Equipment

Dispersions of the PEI-PAA IPEC (Q = [PEI]/[PAA] = 3) containing Ag^+^ ions (see above) were irradiated using an OUFL-01 emitter (DKBU-7 lamp, effective radiation range: 180–275 nm) with constant stirring. The distance from the UV source to the flask containing 30 mL of the solution was 3 cm. The structure of the nanocomposite material was studied using the transmission microscope “Leo-912 AB OMEGA” with a resolution of 0.3 nm. Data on the size and spatial distribution of the nanoparticles were obtained from the analysis of about 150–300 objects in the TEM images. UV-Vis spectra and turbidimetric data were measured using a Perkin Elmer Lambda 9 spectrometer with an optical range of 200–900 nm (Überlingen, Germany). For measuring turbidity, portions of the guest polyelectrolyte (GPE) solutions were successively added to a solution of the host polyelectrolyte (HPE) with an interval of 1 min between titrant additions. Measurements were performed under constant stirring at room temperature directly in a quartz cuvette.

### 2.3. Antibacterial Tests

*Staphylococcus aureus* (strain ATCC 25923, Gram-positive) and *Pseudomonas aeruginosa* (strain ATCC 27853, Gram-negative) were used for the assessment of the antimicrobial activity of the IPEC, IPEC-Ag^1+^, and IPEC-AgNPs. The bacteria were cultured on Tryptic soy broth (TSB, Liofilchem, Roseto degli Abruzzi, Italy) and incubated at 37 °C until reaching a concentration of 1 × 10^6^ CFU/mL. For the flow cytometry test, 10 µL of the IPEC, IPEC-Ag^1+^, or IPEC-AgNPs was added to 90 µL of bacterial culture. A control sample was prepared by mixing 10 µL of the TSB with 90 µL of bacterial culture. All samples were incubated at 37 °C for 20 h. The LIVE/DEAD™ BacLight™ Bacterial Viability and Counting Kit (Thermo Fisher Scientific, Waltham, MA, USA) was used for the assessment of bacteria viability. An aliquot of 987 µL of 0.85% NaCl was mixed with 1.5 µL of red fluorescent propidium iodide (PI) stain, 1.5 µL of green fluorescent (SYTO 9) stain, and 5 µL of bacterial culture. The reaction mixture was incubated in the dark at room temperature for 15 min. After the addition of 5 µL of the microsphere standard, the well-mixed sample was analyzed by flow cytometry with CytoFLEX™ (Beckman Coulter, Bray, CA, USA). The results were presented as a percentage of the live bacteria of their live number in the control sample according to the following equation:

$$Bacteria\;viability\;\left(BV\right)=\frac{Count\;of\;live\;cells\;in\;the\;sample}{Count\;of\;live\;cells\;in\;the\;control}\times 100\%$$

The measurements were carried out in triplicate. The STATISTICA 10.0 software was used for the statistical analysis. The results were calculated as means ± SD. Significant differences were calculated by one-way ANOVA followed by Tukey’s HSD test. Differences with *p*-values < 0.05 were considered statistically significant.

## 3. Results and Discussion

### 3.1. Synthesis of IPEC and IPEC-Ag1^+^ Conjugates

**Figure 1:**
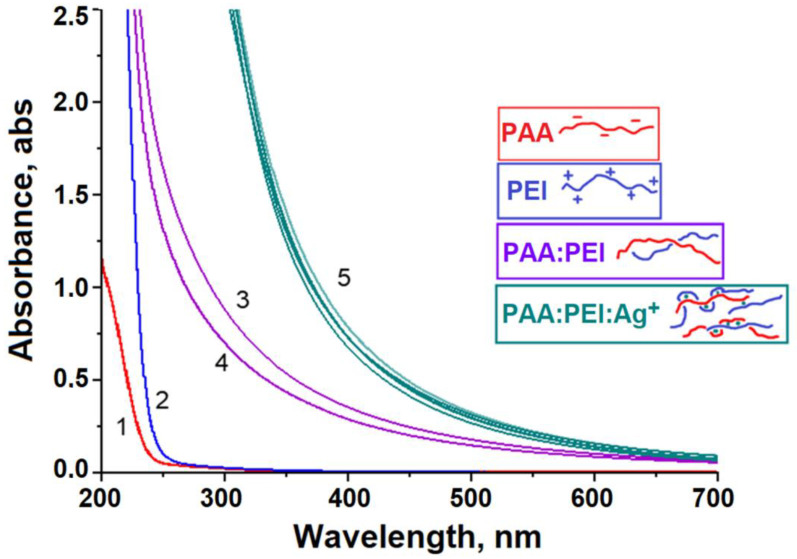
UV–visible spectra of 0.2 wt% aqueous solutions of (1) PAA at pH = 6; (2) PEI at pH =6; (3) freshly prepared IPEC PAA-PEI (Q = 3); (4) IPEC PAA-PEI (Q = 3) after 2 weeks kept at pH = 6; and (5) IPEC PAA-PEI (Q = 3) with silver ions (with a molar ratio of amine groups to silver ions of 1/10) without light sources after the addition of silver nitrate, after 60 min, 120 min, 1 day, 1 week, and 2 weeks.

**Figure 2:**
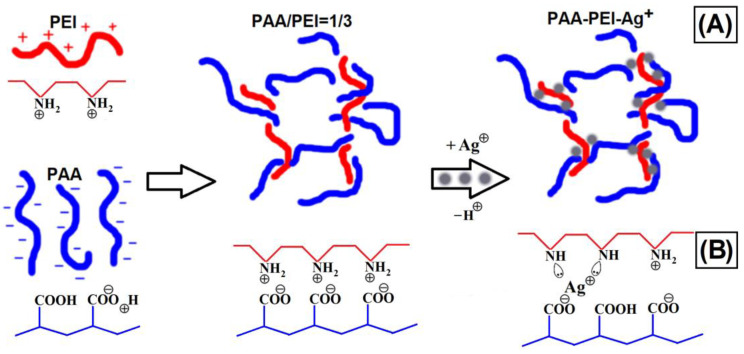
Formation of the interpolyelectrolyte complex PAA-PEI and the ternary metal–polymer complex PAA-PEI-Ag^+^ (**A**). Proposed structure of the coordination sphere of Ag^+^ ions (**B**).

Figure 1 shows the UV-Vis spectra of four aqueous solutions: anionic PAA, cationic PEI, the binary PEI-PAA mixture, and the ternary PEI-PAA-Ag^1+^ mixture. Solutions of individual polymers, PAA (curve 1) and PEI (curve 2), are transparent in the visible and near UV (250–400 nm) ranges of the spectrum, while a solution of the PEI-PAA IPEC with a molar ratio of cationic-to-anionic units Q = [PEI]/[PAA] = 3 demonstrates high extinction in these ranges (curve 3). This fact reflects the formation of scattering IPEC particles stabilized by multiple salt bonds between the cationic and anionic units of both polymers (Figure 2A). The absorption curve profile for the binary PEI-PAA IPEC solution did not change over two weeks (cf. curves 3 and 4), indicating the aggregation stability of the IPEC particles during this period of time. The size of the PEI-PAA IPEC particles measured with dynamic light scattering was approx. 230 nm with a PDI of 0.2 (more detailed information in Supplementary Materials Table S1).

To prepare the PEI-PAA-Ag^+^ metal–polymer complex, a solution of AgNO_3_ was added to the PEI-PAA IPEC dispersions (Q = 3) so that the ratio of cationic units of the PEI to Ag^+^ ions was 10/1. Such a content of metal ions did not exceed the sorption capacity of the IPEC parts of the non-stoichiometric complexes. The UV-Vis spectrum for this system is described by curve 5 (Figure 1). Higher extinction indicated the binding of the silver ions to the IPEC and the formation of ternary PEI-PAA-Ag^1+^ conjugates. The size of the particles of PEI-PAA Ag^+^ was approx. 200 nm with a PDI of 0.2 (more detailed information in Supplementary Materials Table S1), which was comparable to the particles of the PEI-PAA IPEC.

The complexation of metal ions with binary IPECs has been intensively studied previously [23]. In particular, it has been shown that metal ions are capable of being incorporated between the cationic and anionic polymer chains, thus forming a “sandwich” structure, which increases the stability of ternary IPEC-Me^n+^ conjugates. Along this line, the formation of the ternary PEI-PAA-Ag^1+^ “sandwich” conjugates was expected in our case as well (Figure 2B). The metal–polymer complexes were obtained at pH 6 because, in acidic media, the efficiency of the complexation of silver ions with functional groups is suppressed due to the competition between the processes of metal ion binding and the protonation of the functional groups of IPECs (Figure 2B). Indeed, the analysis of the UV–visible spectra showed a decrease in the content of ions bound by PEI and PAA macromolecules (Supplementary File, Figure S1) due to the transition to aquacomplexes. The high turbidity of PEI-PAA dispersions does not allow a reliable investigation of the complexation of silver ions. However, the formation of triple metal–polymer complexes in the case of copper ions was previously revealed by EPR spectroscopy [23]. Therefore, for silver ions, it is logical to propose the structure shown in Figure 2B, according to which the silver ion is coordinated with two nitrogen-containing groups and forms an ionic bond with the carboxylate group (Figure 2B). It should be noted that at pH 6, the degree of protonation of PEI units is 0.41, and the degree of dissociation of carboxylate groups is 0.44 [8] (Supplementary File, Figure S2). Therefore, such conditions ensure the effective formation of the IPEC dispersion. According to the data of turbidimetry, both the PEI-PAA IPEC and the PEI-PAA-Ag^+^ metal–polymer complex remained stable for at least a week (cf. curves 5 and 6 in Figure 1).

### 3.2. Synthesis of Ternary PEI-PAA-Ag Nanoparticle Conjugates

**Figure 3:**
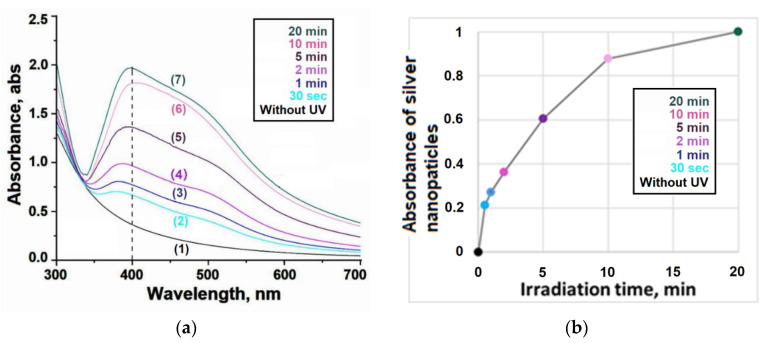
UV–visible spectra (**a**) of 0.2 wt% aqueous solutions of IPEC (Q = [PEI]/[PAA] = 3) with silver ions; effect of UV irradiation time (**b**) on nanoparticle formation.

To prepare the silver nanoparticles, dispersions of the ternary PEI-PAA-Ag^1+^ conjugates were subjected to UV irradiation. During the exposure, a change in the color of the samples was noticed from initially colorless to violet-red, which qualitatively indicated the formation of colloidal metal (silver nanoparticles). Figure 3a shows the time-dependent transformations of the UV-Vis spectrum of the ternary system. The plot describing the conversion of Ag^1+^ ions into neutral Ag nanoparticles is shown in Figure 3b. A 100% yield of nanoparticles was achieved within 20 min.

The high extinction in the visible region was due to the effect of the surface plasmon resonance of silver nanoparticles. The spectra of the AgNPs show two peaks with maxima at wavelengths of ca. 380 and 500 nm. The observed shape of the spectra may indicate the presence of closely spaced AgNPs. Generally speaking, plasmon–plasmon interactions between closely located nanoparticles can lead to the broadening of the SPR bands and the appearance of additional peaks [28,29]. Indeed, the formation of AgNP aggregates can be stimulated by the polymer matrix. Meanwhile, the plasmon spectra of Ag particles with sizes larger than ~100 nm may also exhibit two distinct peaks corresponding to the dipole and quadruple modes [30].

**Figure 4:**
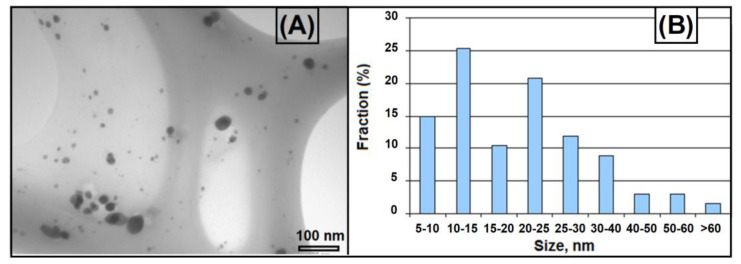
Micrography (**A**) and histogram of size distribution of nanoparticles (**B**) of UV–irradiated PAA–PEI–Ag+ complexes.

The electron transmission microscopy data for the irradiated ternary PEI-PAA-Ag^1+^ conjugates are in agreement with the spectrophotometric results. The micrographs of the samples demonstrate a wide range of particles, the size of which varied from 5 nanometers to 65 nm (Figure 4). It is noteworthy that the nanoparticles tended to form associates. Previously, silver nanoparticles with sizes of less than 25 nm were obtained by X-ray irradiation in the dispersion of PAA-PEI complexes [8]. Thus, with the photochemical method, the ratio of nucleation/growth processes was significantly higher compared with the use of ionizing irradiation. The photolysis of PEI-PAA-Ag^+^ complexes proceeds by electron transfer from COO^−^ or NH groups to Ag^+^ ions, which leads to the formation of Ag^0^ atoms. Subsequent reactions involve the formation of small, charged silver clusters that are reduced and coalesce into NPs.

### 3.3. Antimicrobial Activity of Polymer and Polymer–Metal Formulations

The above-described formulations, the aqueous solutions of the PEI-PAA IPEC, the ternary PEI-PAA-Ag^1+^ complex, and the ternary PEI-PAA-AgNP conjugate, were used to test their antimicrobial activity against the Gram-positive bacteria *Staphylococcus aureus* and the Gram-negative bacteria *Pseudomonas aeruginosa*. The polymer formulations were added to suspensions of cells, the mixtures were incubated for 20 h, and a portion of the surviving cells were quantified using flow cytometry. The duration of 20 h provided the prolonged contact of the cells with the antimicrobial agent, which was expected to produce a pronounced toxic effect. The results are shown in Table 1.

As follows from the data in Table 1, the PEI-PAA IPEC had no significant effect on any of the cell types: in both cases, 30% or more of the cells survived after 20 h. In other words, the binary PEI-PAA IPEC did not work as a biocidal formulation at a 0.2 wt% concentration. Free cationic PEI groups in the binary IPEC were not sufficient to exhibit a biocidal effect.

The addition of a silver salt to the PEI-PAA IPEC and the formation of a ternary PEI-PAA-Ag^1+^ complex resulted in a composition capable of killing the Gram-positive bacteria *S. aureus* but was ineffective against the Gram-negative bacteria *P. aeruginosa*. Further transformation of Ag^1+^ ions into Ag nanoparticles resulted in a strong biocidal effect against both types of bacteria, with the number of surviving cells not exceeding 0.03%.

During UV irradiation, all the Ag^1+^ ions were reduced and formed Ag nanoparticles, as shown in Figure 3b. Therefore, the silver content in PEI-PAA-Ag^1+^ and PEI-PAA-AgNP was the same. If so, both formulations should have demonstrated the same antimicrobial effect. This was true for the Gram-positive bacteria *S. aureus* but not for the Gram-negative bacteria *P. aeruginosa* (Table 1).

## 4. Conclusions

Silver compounds are toxic to microbes. Despite their pronounced biocidal effect, silver ions can be rapidly inactivated by various environmental substances. The silver content in the ternary PEI-PAA-Ag^1+^ complex and PEI-PAA-AgNP conjugate was sufficient to kill the Gram-positive bacteria *S. aureus*. The antimicrobial effect of silver ions is influenced by the structure of the cell shell of microorganisms [18]. Gram-negative bacteria contain lipopolysaccharides (LPSs) in the cell membrane, which contribute to the structural integrity of the membrane, as well as protecting the membrane. The Gram-negative bacteria *P. aeruginosa* are more resistant to bactericidal agents, and, therefore, more agents are required to kill these bacteria [16]. Probably, the strong “sandwich” PEI-PAA-Ag^1+^ complex did not provide a lethal Ag^1+^ concentration, whereas the silver nanoparticles in the PEI-PAA-AgNP conjugate were capable of destroying bacteria. Silver nanoparticles are generally more effective than ions [31,32,33,34,35] due to a combination of the release of Ag^1+^ ions from nanoparticles and the direct interaction of nanoparticles with cell membranes [13,32,36,37,38,39,40,41]. Gram-positive microorganisms often have less sensitivity to silver colloids in comparison with Gram-negative microorganisms [12,13,14,16,19]. Nevertheless, silver nanoparticles with sizes of 10–30 nm immobilized in the PEI-PAA dispersion ensured the effective losses of both *S. Aureus* and *P. aeruginosa* under experimental conditions.

The results obtained in this work show that PEI-PAA IPECs are a universal basis for the production of antibacterial formulations with silver ions and nanoparticles, which is relevant for the development of preparations with the controlled release of biocidal agents. UV irradiation led to the production of nanocomposites without impurities directly in the dispersions of the metal–polymer complexes. The nanocomposites obtained by the photolysis of dispersions of PEI-PAA complexes containing silver ions had a pronounced biocidal effect against both Gram-positive and Gram-negative bacteria.

## Figures and Tables

**Table 1:** Results of bacterial viability (BV) assessment.

Sample ^1^	BV, %	
	*S. aureus*	*P. aeruginosa*
1	2	3
PEI/PAA	79 ± 7	38 ± 11
PEI/PAA/Ag^1+^	0.04 ± 0.01	48 ± 7
PEI/PAA/AgNP	0.03 ± 0.01	N.D.

(^1^)—Polymer conc. 0.2 wt%; Ag conc. 0.065 wt%; 37 °C. N.D.: not detected. Flow cytogram results are given in Supplementary Files in Figure S3 and S4.

## Data Availability

Data is contained within the article or supplementary material.

## Supplementary Materials

The following supporting information can be downloaded at: https://www.mdpi.com/article/10.3390/polym16192842/s1, Figure S1: UV-Vis spectra solutions of PEI (a); PAA (b) with Ag+ ions at pH = 6.0 and pH = 2.5; Figure S2: Dependence of the degree of protonation PEI (αPEI) on pH (a), dependence of the degree of dissociation PAA (αPAA) on pH; Figure S3: Flow cytograms demonstrating antimicrobial activity against *S. aureus* after 20 h of incubation; Figure S4: Flow cytograms demonstrating antimicrobial activity against *P. aeruginosa* after 20 h of incubation; Table S1: DLS data for interpolyelectrolyte and metal polymer complexes of PEI–PAA.
