# Supplementary material for: Preparation of Antimicrobial Agents: From Interpolyelectrolyte Complexes to Silver-Containing Metal–Polymer Complexes and Nanocomposites
Source: Polymers (Basel). 2024 Oct 8;16(19):2842. doi: 10.3390/polym16192842 (PMC11478344; doi:10.3390/polym16192842)
Supplement: Supplementary file 1 [file polymers-16-02842-s001.zip › polymers-3208978-supplementary.pdf]

Supplementary Materials

Preparation of Antimicrobial Agents: From Interpolyelectrolyte Complexes to Silver-Containing Metal–Polymer Complexes and Nanocomposites

Dmitry I. Klimov [1,2], Alexey A. Zharikov [1], Elena A. Zezina [1], Elena A. Kotenkova [3], Elena V. Zaiko [3], Dagmara S. Bataeva [3], Anastasia A. Semenova [3], Yulia K. Yushina [3], Aleksander A. Yaroslavov [1] and Alexey A. Zezin [1,2,*]

1 Department of Chemistry, M.V. Lomonosov Moscow State University, Leninskie gory 1-3, Moscow 119991, Russia; klimovdi@ispm.ru (D.I.K.); garikov-aleksey@mail.ru (A.A.Z.); zezi-len@yandex.ru (E.A.Z.); yaroslav@belozersky.msu.ru (A.A.Y.)
2 Enikolopov Institute of Synthetic Polymeric Materials, Russian Academy of Sciences, Profsoyuznaya st. 70, Moscow 117393, Russia
3 V.M. Gorbatov Federal Research Center for Food Systems, Talalikhina st., 26, Moscow 109316, Russia; lazovlena92@yandex.ru (E.A.K.); e.zaiko@fncps.ru (E.V.Z.); d.bataeva@fncps.ru (D.S.B.); a.semenova@fncps.ru (A.A.S.); yu.yushina@fncps.ru (Y.K.Y.)
* Correspondence: aazezin@yandex.ru

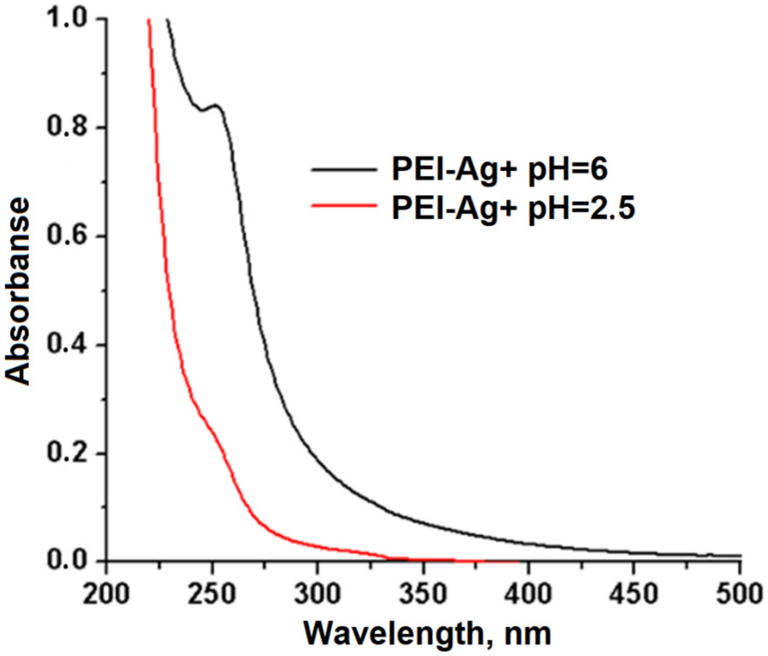

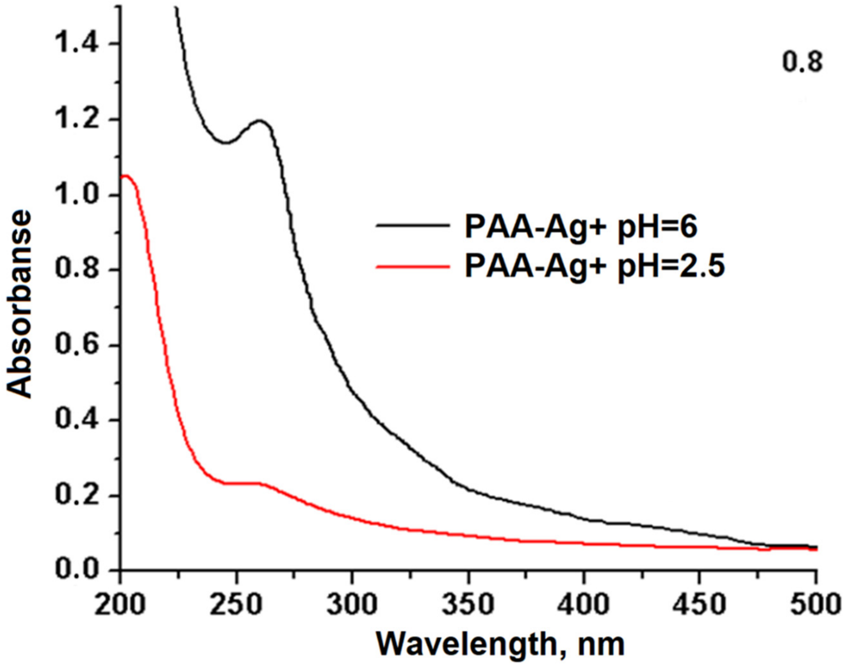

Figure S1. UV-Vis spectra solutions of PEI (a); PAA (b) with Ag^{+} ions at pH=6.0 and pH=2.5. Increase of acidity lead to dramatically decrease of the intensity of these signals, which indicates that most silver ions are in the form of aquacomplexes.

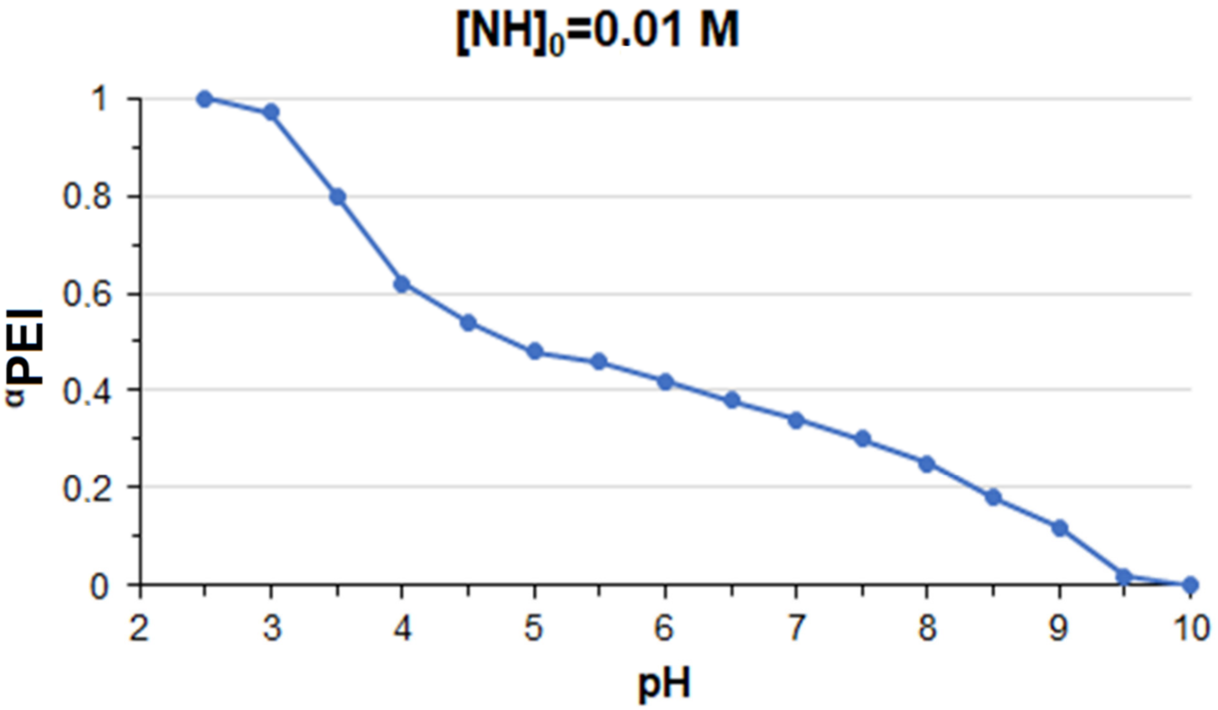

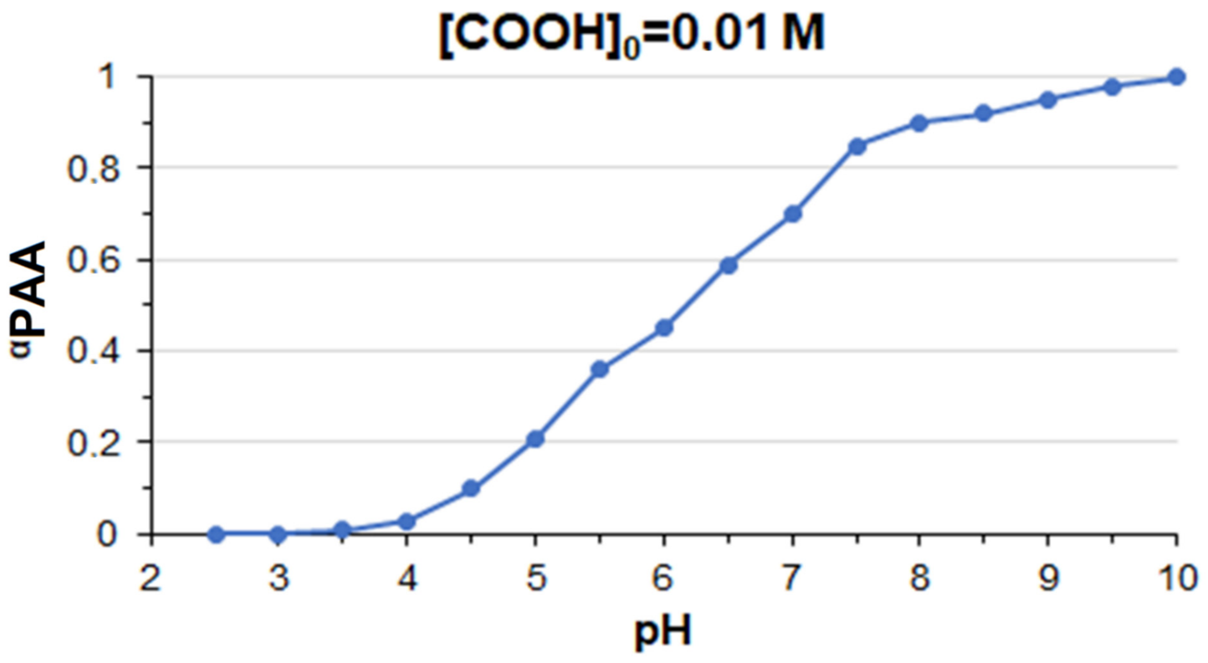

Figure S2. Dependence of the degree of protonation PEI (αPEI) on p◎ (a), dependence of the degree of dissociation PAA (αPAA) on p◎ . Obviously, the maximum number of salt bonds will occur at those p◎ values at which both polymer components carry a significant charge, that is, at 5 ≤ p◎ ≤ 7.

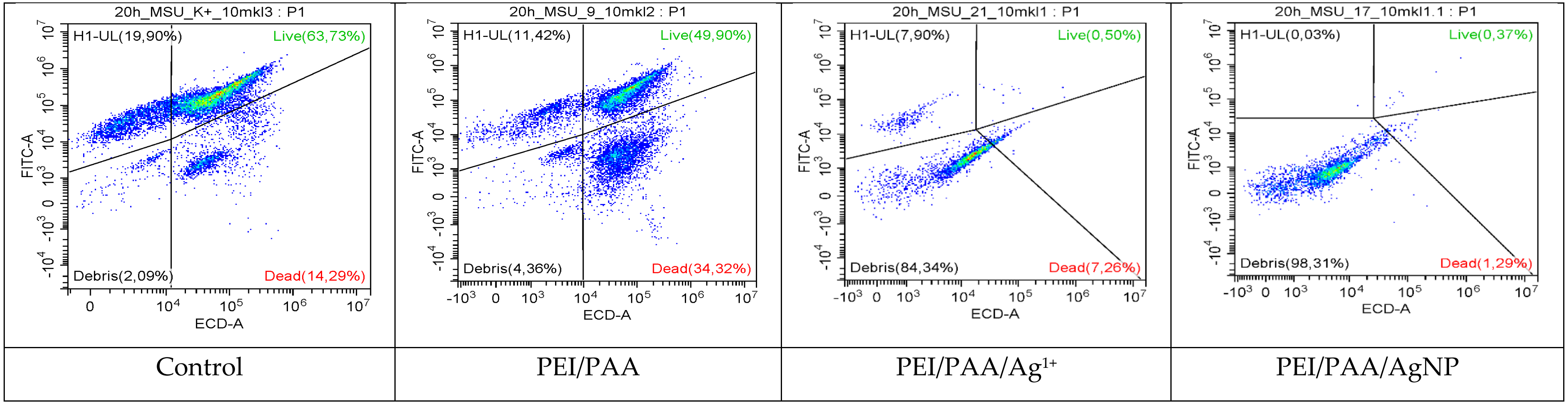

Figure S3. Flow cytograms demonstrating antimicrobial activity against *S. aureus* after 20 hours of incubation.

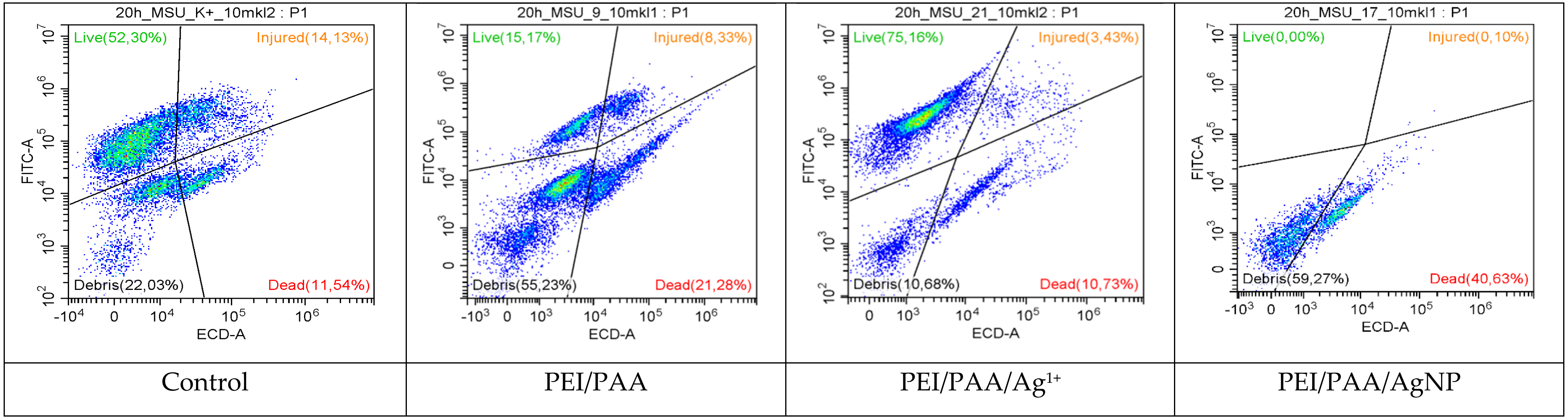

Figure S4. Flow cytograms demonstrating antimicrobial activity against *P. aeruginosa* after 20 hours of incubation.

Table S1. DLS data for interpolyelectrolyte and metal polymer complexes of PEI – PAA.

System	Def, nm	PDI	EPM, (μm/s)/(V/cm)	Zp, mV
PEI-PAA	230	0.2	+2.01 ± 0.13	+25.68 ± 1.69
PEI-PAA-Ag^{+}	200	0.2	+1.78 ± 0.12	+22.75 ± 1.52

Particles of interpolyelectrolyte complexes and metal polymer complexes of branched PEI ((Mw = 60,000) and linear PAA ((Mw = 100,000) are formed with close sizes and a relatively wide size distribution.
